# p27^Kip1^ Directly Represses *Sox2* during Embryonic Stem Cell Differentiation

**DOI:** 10.1016/j.stem.2012.09.014

**Published:** 2012-12-07

**Authors:** Han Li, Manuel Collado, Aranzazu Villasante, Ander Matheu, Cian J. Lynch, Marta Cañamero, Karine Rizzoti, Carmen Carneiro, Gloria Martínez, Anxo Vidal, Robin Lovell-Badge, Manuel Serrano

**Affiliations:** 1 Tumor Suppression Group, Spanish National Cancer Research Centre (CNIO), Madrid, E28029, Spain; 2 Histopathology Unit, Spanish National Cancer Research Centre (CNIO), Madrid, E28029, Spain; 3 Division of Stem Cell Biology and Developmental Genetics, MRC National Institute for Medical Research, Mill Hill, London NW7 1AA, UK; 4 Departamento de Fisioloxia, Facultade de Medicina, Universidade de Santiago de Compostela, Instituto de Investigaciones Sanitarias (IDIS), Santiago de Compostela, E15782, Spain

## Abstract

The mechanisms responsible for the transcriptional silencing of pluripotency genes in differentiated cells are poorly understood. We have observed that cells lacking the tumor suppressor p27 can be reprogrammed into induced pluripotent stem cells (iPSCs) in the absence of ectopic *Sox2*. Interestingly, cells and tissues from *p27* null mice, including brain, lung, and retina, present an elevated basal expression of *Sox2*, suggesting that p27 contributes to the repression of *Sox2*. Furthermore, *p27* null iPSCs fail to fully repress *Sox2* upon differentiation. Mechanistically, we have found that upon differentiation p27 associates to the *SRR2* enhancer of the *Sox2* gene together with a p130-E2F4-SIN3A repressive complex. Finally, *Sox2* haploinsufficiency genetically rescues some of the phenotypes characteristic of *p27* null mice, including gigantism, pituitary hyperplasia, pituitary tumors, and retinal defects. Collectively, these results demonstrate an unprecedented connection between p27 and *Sox2* relevant for reprogramming and cancer and for understanding human pathologies associated with *p27* germline mutations.

## Graphical abstract

**Graphical Abstract:**

**Figure:**
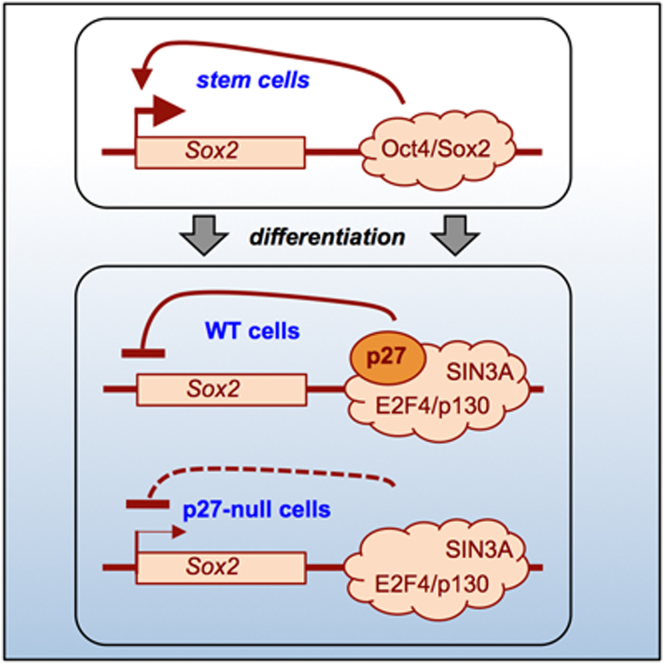

**Highlights:**

► Loss of the tumor suppressor p27 upregulates the pluripotency gene *Sox2* ► Absence of p27 allows reprogramming without ectopic *Sox2* ► p27 associates with a *Sox2* enhancer together with a repressive complex ► SOX2 mediates some of the main phenotypic defects of *p27* null mice

## Teaser

The tumor suppressor p27^Kip1^ has an unexpected role in direct transcriptional regulation of *Sox2* during the differentiation of pluripotent cells.

## Introduction

Differentiated cells can be converted into induced pluripotent stem cells (iPSCs) through the combined action of transcription factors, most notably OCT4, KLF4, and SOX2 (Takahashi and Yamanaka, 2006). Importantly, the mechanisms involved in this process might provide clues about the molecular mechanisms governing stem cell biology and cancer. Recently, we and others have shown that tumor suppressors, such as those encoded by the *p53* gene and the *Ink4a/Arf* locus, oppose reprogramming and limit the efficiency of the process (Banito et al., 2009; Hong et al., 2009; Kawamura et al., 2009; Li et al., 2009; Marión et al., 2009; Utikal et al., 2009; Zhao et al., 2008).

The tumor suppressor p27^Kip1^ binds and inhibits multiple cyclin-dependent kinases (Besson et al., 2008). Importantly, low protein levels of p27 constitute a poor prognosis marker for several types of cancer (Chu et al., 2008) and germline mutations of the *p27* gene (also known as *CDKN1B*) are responsible for a subset of human multiple endocrine neoplasia (MEN) syndromes, notably characterized by pituitary tumors (Marinoni and Pellegata, 2011; Vandeva et al., 2010). The cyclin-dependent kinase 2 (CDK2) is one of the main CDKs inhibited by p27 (Besson et al., 2008). Paradoxically, however, the main phenotypes of *p27* null mice, namely, increased body size, organ hyperplasia, pituitary tumors, and retinal dysplasia (Fero et al., 1996; Kiyokawa et al., 1996; Nakayama et al., 1996), are not rescued by concomitant deletion of *Cdk2*, thus suggesting that these *p27* null phenotypes are not primarily caused by uncontrolled CDK2 activity (Aleem et al., 2005; Martín et al., 2005).

In the context of investigating the role of tumor suppressors during reprogramming, we studied *p27* null cells and we noticed that these cells can be reprogrammed into iPSCs without ectopic expression of *Sox2*. This observation led us to explore the potential link between these two previously unrelated proteins, p27 and SOX2.

## Results

### Cells Lacking *p27* Express Higher Levels of *Sox2* and Can Be Reprogrammed without Ectopic *Sox2*

**Figure 1:**
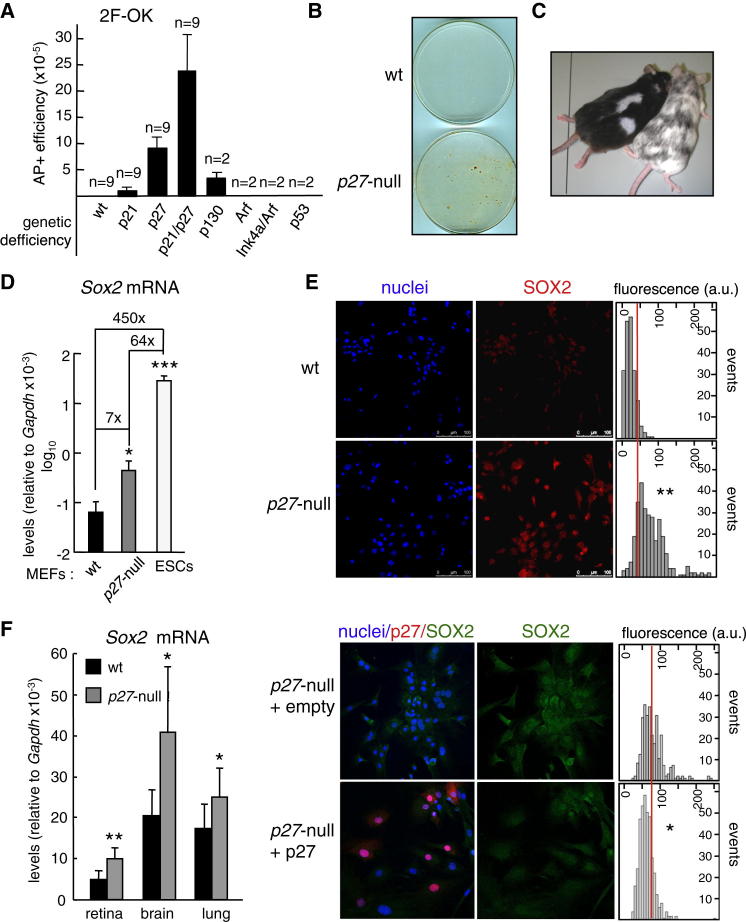
Absence of *p27* Allows Two-Factor (*Oct4* and *Klf4*) Reprogramming (A) Two-factor (*Oct4* and *Klf4*, 2F-OK) reprogramming of primary MEFs of the indicated genotype. Efficiency is measured as the number of alkaline-phosphatase-positive (AP^+^) colonies relative to the total number of infected cells. n values correspond to independent MEF isolates. (B) Representative picture of AP stained plates. (C) Picture of chimeric mice generated from *p27* null/2F-OK iPSCs (black-C57BL6 genetic background) after microinjection into albino-C57BL6 blastocysts. (D) *Sox2* mRNA levels in WT (n = 3) and *p27* null (n = 5) MEFs and ESCs. mRNA levels were determined by qRT-PCR. (E) Upper panel: representative picture of SOX2 immunofluorescence in WT and *p27* null MEFs and quantification of the immunofluorescence corresponding to one experiment. A total of two experiments were performed, each with different MEF isolates, with similar results obtained in both of them. Lower panel: representative picture of p27 and SOX2 immunofluorescence in *p27* null MEFs infected with empty vector or with pBabe-p27. A total of three independent experiments were performed, each with different MEF isolates, and similar results were obtained in the three of them. The average ± SD of each distribution was compared with its corresponding control using the Student's t test. (F) *Sox2* mRNA levels in WT (n = 6) and *p27* null (n = 10) mice (∼1 year old). All data correspond to the average ± SD. Statistical significance was assessed by two-tailed Student's t test: ^∗∗∗^p < 0.001; ^∗∗^p < 0.01; ^∗^p < 0.05. See also Figure S1.

While investigating the effect of tumor suppressor genes on the process of reprogramming to induced pluripotent stem cells (iPSCs) by the three Yamanaka factors (*Oct4*, *Klf4*, and *Sox2*) (Takahashi and Yamanaka, 2006), we tested the three possible combinations of two factors (abbreviated as 2F-OK, 2F-OS, and 2F-KS in reference to Oct4, Klf4, and Sox2) in a series of primary mouse embryo fibroblasts (MEFs) lacking cell cycle regulators and tumor suppressors. After repeated attempts, we were unable to obtain alkaline-phosphatase-positive (AP^+^) colonies in any of the tested MEFs using 2F-OS or 2F-KS. Interestingly, however, *p27* null MEFs and, to a lesser extent, *p130* null MEFs gave rise to AP^+^ colonies with 2F-OK (Figures 1A and 1B). Absence of the p27-related protein p21 also produced AP^+^ colonies and further increased the number of AP^+^ colonies when combined with *p27* deficiency, thus suggesting some degree of functional redundancy between p27 and p21. In all these MEFs, the emergence of visible AP^+^ colonies was delayed compared to the standard three-factor cocktail (3F-OKS) (4 weeks versus 2 weeks) and the average efficiency was about 100-fold lower (9 × 10^−5^ in *p27* null/2F-OK versus 8 × 10^−3^ in WT/3F-OKS). In contrast to this, WT MEFs or MEFs deficient in *p53*, *Arf*, or *Ink4a/Arf* could not be reprogrammed by 2F-OK despite the fact that these cells are reprogrammed with very high efficiency by 3F-OKS (Banito et al., 2009; Hong et al., 2009; Kawamura et al., 2009; Li et al., 2009; Marión et al., 2009; Utikal et al., 2009; Zhao et al., 2008). Also, absence of *p27* had a modest stimulatory effect on 3F-OKS reprogramming (Figure S1A available online). Together, these observations suggest that the absence of *p27* selectively renders cells susceptible to reprogramming in the absence of ectopic *Sox2*. The *p27* null/2F-OK AP^+^ colonies were confirmed to be bona fide iPSCs based on their expression of endogenous pluripotency genes (*Nanog*, *Sox2*, and *Oct4*; Figure S1B), production of teratomas (Figure S1C), and efficient contribution to chimeric mice (Figure 1C). To further validate the use of alkaline phosphatase as a marker of reprogramming under our experimental conditions (most notably characterized by the absence of ectopic c-Myc and by the use of serum-free medium), we obtained WT and *p27* null MEFs carrying a transgenic *GFP* reporter under the *Sox2* promoter (D'Amour and Gage, 2003), and we observed that >90% of the AP^+^ colonies were GFP^+^. All together, these results indicate that the absence of *p27* eliminates the absolute requirement for ectopic *Sox2* in reprogramming.

Mouse fibroblasts express low, but detectable, levels of *Sox2* (Eminli et al., 2008), and therefore, we wondered whether *p27* deficiency affected *Sox2* expression. Indeed, *p27* null MEFs had a significant increase in *Sox2* mRNA levels compared to WT controls (7-fold), although these levels were still about 64-fold lower than in embryonic stem cells (ESCs) (Figure 1D). We also detected SOX2 protein by immunofluorescence. Consistent with the mRNA data, quantitative image analyses indicated that SOX2 protein levels were globally increased in *p27* null MEFs (Figure 1E). Of note, the distribution of SOX2 fluorescence intensity in *p27* null MEFs is broad, and therefore, it is conceivable that only those *p27* null cells with the highest SOX2 levels are the ones susceptible of 2F-OK reprogramming. It is also worth mentioning that WT and *p27* null MEFs have the same proliferative rate (Coats et al., 1999), thus implying that their different SOX2 levels are not secondary to a different proliferative activity. We tested whether the increased levels of SOX2 observed in *p27* null MEFs could be reverted by ectopic overexpression of p27. Interestingly, quantitative immunofluorescence of p27-overexpressing *p27* null MEFs indicated a downregulation of SOX2 levels (Figure 1E). Finally, we also observed higher *Sox2* mRNA levels in the retina, brain, and lung of adult *p27* null mice (Figure 1F). Together, these data indicate that p27 contributes to the silencing of *Sox2* in differentiated cells and tissues.

### *Sox2* Expression Is Repressed by p27

**Figure 2:**
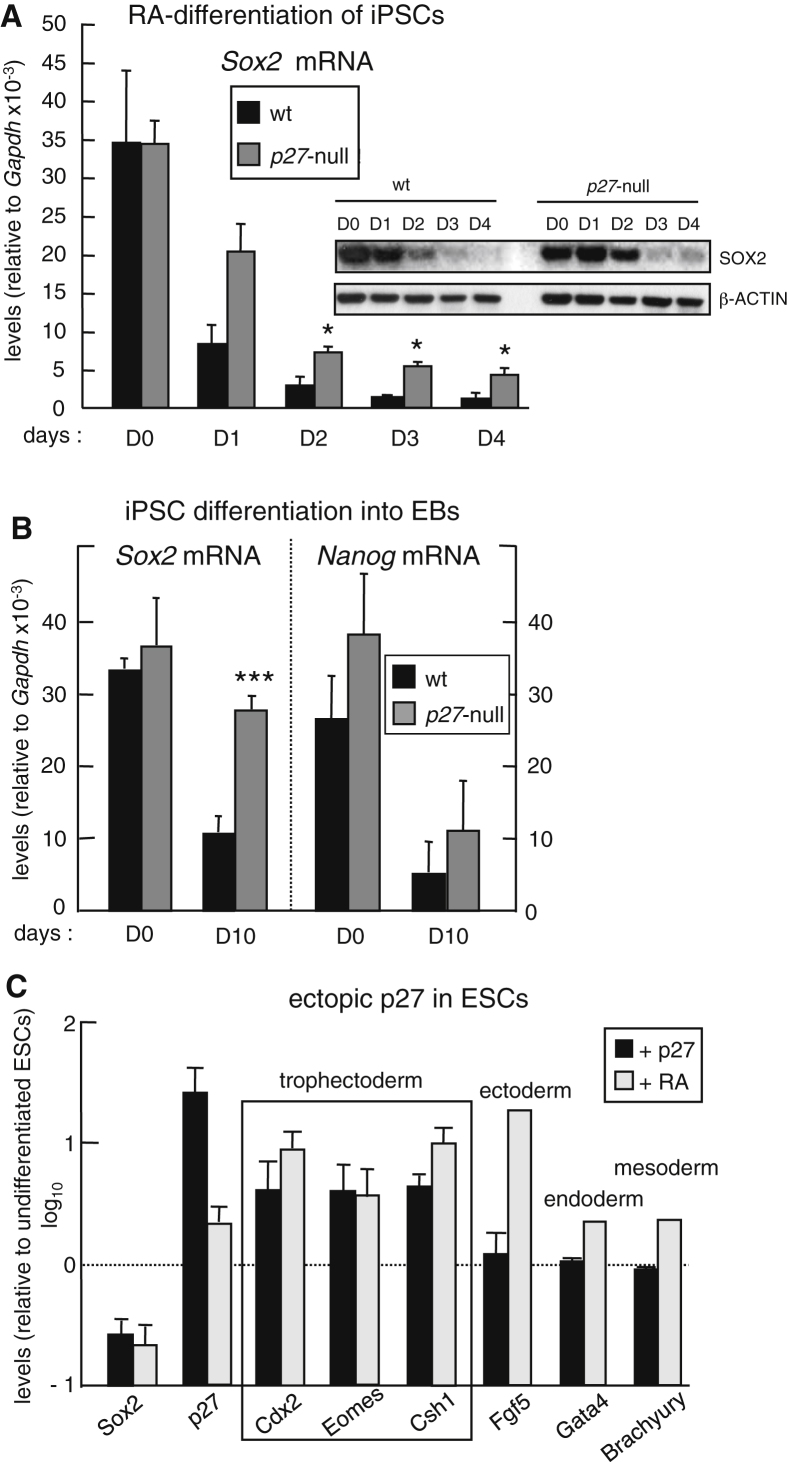
p27 Represses *Sox2* Expression (A) *Sox2* mRNA and protein levels in iPSCs undergoing in vitro differentiation by the addition of retinoic acid (RA) in the absence of LIF for the indicated number of days. mRNA values correspond to the average ± SD. (n = 6 independent clones per genotype.) (B) *Sox2* and *Nanog* mRNA levels in iPSCs before and after 10 days of induction of embryoid bodies (EBs). mRNA values correspond to the average ± SD. (n = 6 independent clones per genotype.) (C) *Sox2* and *p27* mRNA levels in ESCs after infection (3 days) with an empty vector or with a plasmid overexpressing p27 (pMSCV-p27). ESCs at day 4 of the RA-differentiation protocol were used as a differentiation control. Trophectoderm markers and markers for other lineages were tested. In the case of ESCs infected with pMSCV-p27, values are relative to ESCs infected with empty vector. In the case of RA-differentiated ESCs, values are relative to nondifferentiated ESCs. Three independent assays were performed for overexpression of p27 (n = 3). mRNA levels were determined by qRT-PCR. All data correspond to the average ± SD. Statistical significance was assessed by the two-tailed Student's t test: ^∗∗∗^p < 0.01; ^∗^p < 0.05. See also Figure S2.

To further define the repressive role of p27 on *Sox2*, we analyzed the differentiation of pluripotent stem cells upon treatment with retinoic acid (RA). This differentiation protocol efficiently reduces SOX2 protein and mRNA concomitantly with a dramatic upregulation of p27 (Figure S2A). The upregulation of p27 during RA-induced differentiation is in agreement with a previous report on differentiating human embryonic carcinoma cells (Bahrami et al., 2005). We wondered whether the absence of p27 would affect the kinetics of *Sox2* repression. For this, we generated iPSCs derived from WT or *p27* null MEFs after reprogramming them with *Oct4*, *Klf4*, and *Sox2*. The levels of *Sox2* mRNA were similar in undifferentiated WT or *p27* null iPSCs (Figure 2A). However, upon RA-induced differentiation, *Sox2* levels in *p27* null iPSCs were not reduced as efficiently as in WT iPSCs (Figure 2A). Similarly, we tested *Sox2* expression during iPSC differentiation into embryoid bodies (EBs). Again, *Sox2* mRNA levels were abnormally high in *p27*-deficient EBs compared to WT ones (Figure 2B). These observations are in agreement with a previous study reporting numerous abnormalities in EBs from *p27* null ESCs (Bryja et al., 2005). We considered the possibility that the increased *Sox2* levels in *p27* null EBs could reflect a skewed neural differentiation, but the levels of *Nestin* mRNA were similar in WT and *p27* null EBs (Figure S2B). To directly test the repressive activity of p27, we retrovirally transduced ESCs with a pMSCV vector expressing p27, and interestingly, p27 overexpression was able to reduce the levels of *Sox2* to an extent comparable to RA (Figure 2C). Previous studies have demonstrated that *Sox2* null ESCs spontaneously differentiate into trophectoderm-like cells (Masui et al., 2007). We asked whether p27 overexpression in ESCs promotes the trophectoderm-like differentiation characteristic of *Sox2* null ESCs. Indeed, this was the case and p27 overexpression selectively induced trophectoderm markers to a similar extent as RA differentiation, whereas ectoderm, endoderm, and mesoderm markers were not induced by p27 but were induced by RA (Figure 2C). Moreover, p27-overexpressing ESCs produced cells with giant trophoblast-like morphology, while this type of cell was absent in RA-differentiated ESCs (Figure S2C). To extend the generality of these findings to other pluripotent cells, we overexpressed p27 in murine P19 embryonal carcinoma (P19EC) cells at levels that did not affect proliferation and found a significant decrease of *Sox2* mRNA levels (Figure S2D). Together, these results indicate that p27 exerts a repressive effect on *Sox2* that is relevant during differentiation.

### p27 Associates to the *Sox2-SRR2* Enhancer Together with Repressive Complex p130-E2F4-SIN3A

**Figure 3:**
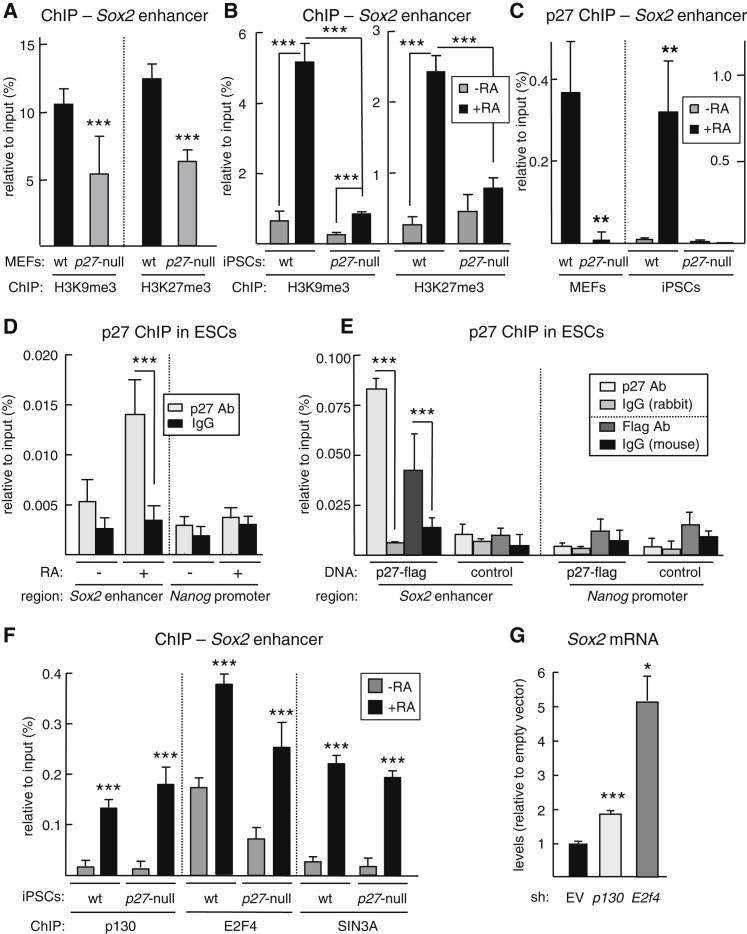
p27 Directly Binds to the *Sox2-SRR2* Enhancer (A) Chromatin immunoprecipitation (ChIP) of H3K9me3 and H3K27me3 in the *Sox2-SRR2* enhancer of WT and *p27* null MEFs. Data correspond to one representative assay from a total of three independent assays, each of them with different MEF isolates. (B) ChIP of the indicated proteins in the *Sox2-SRR2* enhancer of WT and *p27* null iPSCs before and after RA differentiation. Data correspond to one representative assay from a total of two independent assays, each of them with different iPSC clones. (C) ChIP of p27 in the *Sox2-SRR2* enhancer of WT and *p27* null MEFs, and in WT and *p27* null iPSCs before and after RA differentiation. Data correspond to one representative assay from a total of three independent assays, each of them with different MEF isolates and iPSCs clones. (D) ChIP of p27 on the *Sox2-SRR2* enhancer of ESCs before and after RA differentiation. Data correspond to one representative assay from a total of two independent assays. (E) ChIP of p27 in ESCs 2 days after transfection with empty vector (control) or a plasmid expressing flag-tagged p27 (p27-flag). (F) ChIP of the indicated proteins in the *Sox2-SRR2* enhancer before and after RA differentiation of WT and *p27* null iPSCs. (G) *Sox2* mRNA levels in WT MEFs 48 hr after retroviral transduction with sh*p130*, sh*E2f4*, or empty vector (EV). Data correspond to two independent assays (n = 2). All data correspond to the average ± SD. Statistical significance was assessed by the two-tailed Student's t test: ^∗∗∗^p < 0.001; ^∗∗^p < 0.01; ^∗^p < 0.05. See also Figure S3.

The main regulatory element responsible for the expression of *Sox2* in pluripotent stem cells is located ∼4 kb downstream of the single *Sox2* coding exon and it is named *SRR2* (Sikorska et al., 2008; Tomioka et al., 2002). Based on our above observations in MEFs and in differentiating pluripotent cells, we asked whether the presence or absence of p27 had an effect on the repressive epigenetic marks on the *Sox2-SRR2* enhancer. Interestingly, we observed that *p27* null MEFs present lower levels of the H3K9me3 and H3K27me3 repressive marks at the *Sox2-SRR2* enhancer as evidenced by chromatin immunoprecipitation (ChIP) analysis (Figure 3A). Similarly, RA-induced differentiation of WT iPSCs dramatically increased the levels of H3K9me3 and H3K27me3 at the *Sox2-SRR2* enhancer, while these epigenetic marks were modestly increased in *p27* null iPSCs (Figure 3B). These results indicate that the absence of p27 leads to a defective epigenetic remodeling of the *Sox2-SRR2* enhancer both in differentiated cells (MEFs) and during the differentiation of pluripotent cells.

Based on the above data and the recent report that p27 can associate to gene promoters in association with the repressive complex p130-E2F4-SIN3A (Pippa et al., 2012), we hypothesized that p27 might be recruited in this manner to the *Sox2-SRR2* enhancer. To directly test this, we performed ChIP with anti-p27 antibodies in MEFs and we detected p27 associated to the *Sox2-SRR2* enhancer (Figure 3C). As before, we wondered whether this was also the case in differentiating pluripotent cells. Interestingly, RA-induced differentiation of iPSCs was accompanied by a strong recruitment of p27 to the *Sox2-SRR2* enhancer (Figure 3C). To further extend these observations, we used ESCs and, as in the case of iPSCs, p27 was immunoprecipated at the *Sox2-SRR2* enhancer upon RA-induced differentiation, but not at the *Nanog* promoter used here as a control (Figure 3D). We sought additional proof by performing ChIP from ESCs transfected with flag-tagged p27 and we also found p27 bound to the *Sox2-SRR2* enhancer after immunoprecipitation with antibodies against p27 or against the flag tag (Figure 3E). Similar results were obtained in P19EC cells, both upon RA-induced differentiation (Figure S3A) and upon transfection of flag-tagged p27 (Figure S3B).

To examine the presence of a p130-E2F4-SIN3A repressive complex at the *Sox2-SRR2* enhancer, we performed ChIP assays using antibodies against p130, E2F4, and SIN3A in WT or *p27* null iPSCs undergoing RA-induced differentiation. Interestingly, the three proteins were detected in the *Sox2-SRR2* enhancer of RA-differentiated cells regardless of the presence or absence of *p27* (Figure 3F). We also detected binding of p130, E2F4, and SIN3A to the *Sox2-SRR2* enhancer in WT and *p27* null MEFs (Figure S3C) and RA-differentiated P19EC cells (Figure S3D). These results indicate that a repressive p130-E2F4-SIN3A complex is assembled at the *Sox2-SRR2* enhancer upon differentiation and independently of p27.

Finally, we asked whether the direct inhibition of the p130-E2F4-SIN3A complex would also result in derepression of *Sox2*. In agreement with previous reports (Dannenberg et al., 2005), depletion of SIN3A had a dramatic effect on cell viability that precluded us from further examining the effect on *Sox2* expression. Interestingly, however, knockdown of *p130* or *E2f4* with shRNAs resulted in a severe reduction of their expression (Figure S3E) and, importantly, this was accompanied in both cases by a significant upregulation of *Sox2* expression (Figure 3G). In summary, we conclude that p27 associates to the *Sox2-SRR2* enhancer together with the repressive complex p130-E2F4-SIN3A, and together contribute to the repression of *Sox2* upon cell differentiation.

### *Sox2* Heterozygosity Rescues *p27* Deficiency in Mice

**Figure 4:**
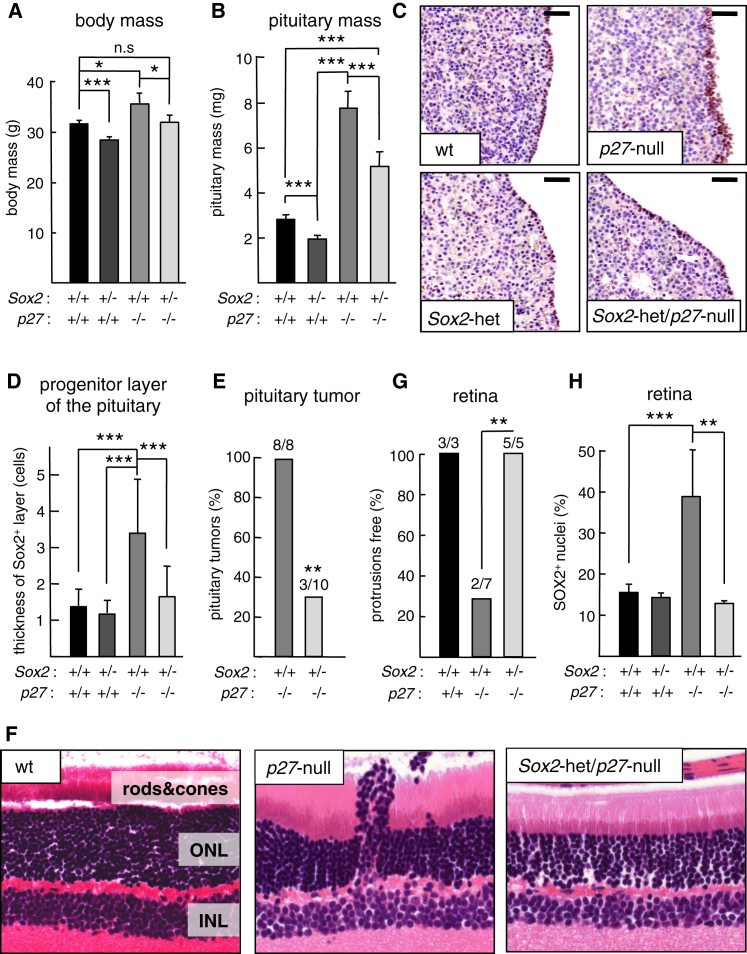
*p27* Null Phenotypes Are Rescued by *Sox2* Haploinsufficiency (A) Body mass of 2-month-old males of the indicated genotypes (n = 3 for WT; n = 6 for *Sox2*-het; n = 5 for *p27*-null; and n = 3 for *Sox2*-het/*p27*-null). (B) Pituitary mass (n = 9 for WT; n = 12 for *Sox2*-het; n = 7 for *p27*-null; and n = 10 for *Sox2*-het/*p27*-null, males and females pooled, 3–6 months old). (C) Representative pictures of the progenitor layer of the pituitary cleft stained with SOX2. Bars correspond to 50 μm. (D) Thickness of the progenitor layer of the pituitary cleft expressed as number of cells (n = 3 for each genotype, males and females pooled, 3–6 months old). (E) Incidence of pituitary adenomas in 3- to 6-month-old mice. (F) Representative pictures of the retina (H&E staining). A focal protrusion is apparent in the *p27* null retina. ONL = outer nuclear layer; INL = inner nuclear layer. (G) Incidence of retinas free of protrusions (n = 3 for WT; n = 7 for *p27*-null; and n = 5 for *Sox2*-het/*p27*-null, males and females pooled, 1 year old). (H) Relative number of SOX2^+^ nuclei in the retina (n = 3 for each genotype, males and females pooled, 1 year old). Data in (A), (B), (D), and (H) correspond to the average ± SD and statistical significance was assessed by the two-tailed Student's t test. Data in (E) and (G) correspond to ratios and statistical significance was assessed by the Fisher's test. ^∗∗∗^p < 0.001; ^∗∗^p < 0.01; ^∗^p < 0.05; n.s., not significant. See also Figure S4.

Based on our above data, we wondered whether the incomplete repression of *Sox2* observed in *p27*-deficient cells and tissues could mediate some of the phenotypes characteristic of *p27* null mice. To evaluate this in a genetic manner, we generated compound *Sox2* heterozygous (*Sox2*-het) and *p27* null mice. *Sox2* null mice are not viable, but *Sox2*-het are viable and display a moderate reduction in body size and hypopituitarism (Kelberman et al., 2006). Interestingly, the characteristic gigantism of *p27* null mice was normalized by deletion of one *Sox2* allele (Figure 4A; Figure S4A), as was also the case for the pituitary mass in young 3- to 6-month-old mice (at this age, *p27* null mice present pituitary hyperplasia, but do not have pituitary tumors yet) (Figure 4B). However, given the fact that *Sox2*-het mice have a modest, but detectable, defect in growth, the above results leave open the possibility that *p27* and *Sox2* simply have opposite effects on growth that balance each other. For this reason, we decided to focus in the progenitor cell layer that surrounds the pituitary cleft and which is formed by SOX2-positive (SOX2^+^) cells (Fauquier et al., 2008; Garcia-Lavandeira et al., 2009; Gleiberman et al., 2008). The progenitor layer in *Sox2*-het pituitaries had the same thickness as in WT pituitaries (Figures 4C and 4D; Figure S4B). Interestingly, the thickness of the progenitor layer was significantly increased in *p27* null mice compared to WT or to *Sox2*-het mice, and this defect was absent in *Sox2*-het/*p27*-null mice (Figures 4C and 4D). The progenitor cells of the pituitary have been proposed to constitute the origin of pituitary adenomas (Gleiberman et al., 2008). In this regard and in line with our above observations, the incidence of pituitary tumors was significantly reduced in *Sox2*-het/*p27*-null mice compared to *p27* null littermates (Figure 4E).

Having established that *Sox2* heterozygosity rescues the gigantism and the pituitary phenotypes of *p27* null mice, we wondered whether the same was true for the retinal defects of *p27* null mice. In agreement with previous reports, the retinas of *Sox2*-het mice were normal (Taranova et al., 2006) (Figure S4C), while *p27* null retinas presented focal protrusions of the outer nuclear layer (ONL) (Nakayama et al., 1996) (Figures 4F and 4G). Interestingly, these protrusions were absent in *Sox2*-het/*p27*-null retinas (Figures 4F and 4G). Also, we observed that *p27* null retinas present an increased abundance of SOX2^+^ nuclei at the inner nuclear layer (INL) and mislocalized SOX2^+^ nuclei in the outer plexiform layer (OPL) (Figure S4C). We quantified the number of SOX2^+^ nuclei in complete retinal sections and confirmed that *p27* null retinas present an excess of SOX2^+^ nuclei and, importantly, we found that this defect was absent in *Sox2*-het/*p27*-null retinas (Figure 4H). We wanted to corroborate the increased abundance of SOX2^+^ nuclei in the absence of *p27*, and for this we used transgenic mice with *GFP* under the control of a *Sox2* promoter region that marks neural multipotent progenitors associated to *Sox2* expression (*Sox2*-*GFP* mice) (D'Amour and Gage, 2003). Reinforcing our above observations, *p27*-het/*Sox2*-*GFP* retinas presented a significant increase in GFP^+^ nuclei when compared with WT/*Sox2*-*GFP* retinas (Figure S4D). Together, we conclude that a decrease in the gene dosage of *Sox2* rescues the main phenotypes associated with *p27* deficiency, namely, gigantism, pituitary hyperplasia, pituitary adenomas, and retinal abnormalities. In addition to these phenotypes, *p27* null mice also have adrenal gland hyperplasias and tumors (pheochromocytomas) and female sterility (Fero et al., 1996; Kiyokawa et al., 1996; Nakayama et al., 1996). We examined WT adrenal glands and *p27* null pheochromocytomas by immunostaining for SOX2, but they were negative and for this reason we did not further pursue this phenotype. Regarding the sterility of *p27* null females, *Sox2*-het/*p27*-null females remained sterile, suggesting that this phenotype is independent of *Sox2*. Collectively, these results provide genetic support to the concept that p27 is a negative regulator of *Sox2* in the pituitary and in the retina.

## Discussion

The mechanisms responsible for the transcriptional silencing of pluripotency genes in differentiated cells are poorly understood. The results reported here demonstrate that the tumor suppressor p27 contributes to the transcriptional repression of *Sox2*. We have observed that the absence of p27 leads to a defective repression of *Sox2* in fibroblasts, lung, retina, and brain, and to a delayed and incomplete silencing of *Sox2* during differentiation of pluripotent cells, including iPSCs, ESCs, and P19EC cells. These observations led us to identify p27 as a transcriptional regulator of *Sox2* together with a repressive complex formed by p130, E2F4, and SIN3A at a critical enhancer responsible for *Sox2* expression. These findings are in line with a recent report describing the capacity of p27 to interact with the p130/E2F4/SIN3A complex and contribute to its transcriptional repressive activity (Pippa et al., 2012). We have found that *p27* deficiency leads to an expansion of SOX2^+^ cells in the progenitor layer of the pituitary and in the retina, which results in pituitary hyperplasia and tumors and morphological defects of the retina. Importantly, these defects are rescued when *p27* deficiency is combined with *Sox2* heterozygosity. In humans, germline mutations in *p27* and *SOX2* also affect the pituitary and the retina. On one hand, loss-of-function mutations in *p27* produce MEN syndrome, notably characterized by pituitary tumors (Marinoni and Pellegata, 2011; Vandeva et al., 2010). On the other hand, loss-of-function mutations in *SOX2* produce syndromes characterized by anophthalmia and hypopituitarism (Engelen et al., 2011; Fantes et al., 2003; Kelberman et al., 2006; Williamson et al., 2006). Our current findings unveil a mechanistic connection between p27 and SOX2, and thereby contribute to our understanding of the molecular basis of the human pathologies associated with the deregulation of these two proteins.

## Experimental Procedures

### Mice

Mice *p27* null (Fero et al., 1996), *Sox2*-het (Avilion et al., 2003), and *Sox2-*promoter*/GFP* transgenic (D'Amour and Gage, 2003) have been previously described. All comparisons were made among mice derived from the same sets of crosses, and they therefore shared the same genetic background. Animal experimentation at the CNIO, Madrid was performed according to protocols approved by the CNIO-ISCIII Ethics Committee for Research and Animal Welfare (CEIyBA) and animal experimentation at the MRC-NIMR, Mill Hill, London was carried out in accordance with the UK Animals (Scientific Procedures) Act 1986.

### ChIP, RNA Quantification, and Protein Analyses

ChIP and quantitative PCR was performed following standard methods (detailed in the Supplemental Experimental Procedures). PCR primer sequences, shRNA encoding plasmids, and other DNA constructs, as well as antibodies and other standard molecular biology methods, are all detailed in Supplemental Experimental Procedures.

### Generation of iPSCs

Reprogramming of primary (passage 2–4) MEFs was performed as previously described by us (Li et al., 2009) using plasmids pMXs-Klf4, pMXs-Sox2, or pMXs-Oct4 (obtained from Addgene and previously described; Takahashi and Yamanaka, 2006). For additional details, see Supplemental Experimental Procedures.

### Differentiation with RA

Differentiation with RA was performed essentially as described (Savatier et al., 1996). ESCs or iPSCs were adapted to grow on gelatin-coated plates (and in the absence of feeder cells). Cells were grown to near confluency in their corresponding complete medium (day 0) and then were trypsinized and seeded at lower density in the absence of LIF for 1 day (day 1). During the following 2 days (days 2 and 3), RA was added at a concentration of 10^−6^ M, and day 4 cells were without LIF and without RA. In the case of P19EC cells, differentiation was induced by addition of RA (10^−6^ M) for 4 days.

### Immunohistochemistry and Immunofluorescence

For immunohistochemical stainings, quantifications were performed on representative fields at the same magnification, on a minimum of three different areas per sample and a minimum of three different samples per genotype. For immunofluorescence, cells were inspected under a Leica TCS-SP5 confocal microscope (AOBS) and analyzed using Definiens Developer XD 1.5 software, under the same exposure conditions. For additional details, see Supplemental Experimental Procedures.

### Statistical Analysis

Unless otherwise specified (Figures 4E and 4G), quantitative data are presented as mean ± SD and significance was assessed by the two-tailed Student's t test.

## Supplemental Information

Document S1. Figures S1–S4 and Supplemental Experimental Procedures

## References

[bib1] Aleem E., Kiyokawa H., Kaldis P. (2005). Cdc2-cyclin E complexes regulate the G1/S phase transition. Nat. Cell Biol..

[bib2] Avilion A.A., Nicolis S.K., Pevny L.H., Perez L., Vivian N., Lovell-Badge R. (2003). Multipotent cell lineages in early mouse development depend on SOX2 function. Genes Dev..

[bib3] Bahrami A.R., Matin M.M., Andrews P.W. (2005). The CDK inhibitor p27 enhances neural differentiation in pluripotent NTERA2 human EC cells, but does not permit differentiation of 2102Ep nullipotent human EC cells. Mech. Dev..

[bib4] Banito A., Rashid S.T., Acosta J.C., Li S., Pereira C.F., Geti I., Pinho S., Silva J.C., Azuara V., Walsh M. (2009). Senescence impairs successful reprogramming to pluripotent stem cells. Genes Dev..

[bib5] Besson A., Dowdy S.F., Roberts J.M. (2008). CDK inhibitors: cell cycle regulators and beyond. Dev. Cell.

[bib6] Bryja V., Cajánek L., Pacherník J., Hall A.C., Horváth V., Dvorák P., Hampl A. (2005). Abnormal development of mouse embryoid bodies lacking p27Kip1 cell cycle regulator. Stem Cells.

[bib7] Chu I.M., Hengst L., Slingerland J.M. (2008). The Cdk inhibitor p27 in human cancer: prognostic potential and relevance to anticancer therapy. Nat. Rev. Cancer.

[bib8] Coats S., Whyte P., Fero M.L., Lacy S., Chung G., Randel E., Firpo E., Roberts J.M. (1999). A new pathway for mitogen-dependent cdk2 regulation uncovered in p27(Kip1)-deficient cells. Curr. Biol..

[bib9] D'Amour K.A., Gage F.H. (2003). Genetic and functional differences between multipotent neural and pluripotent embryonic stem cells. Proc. Natl. Acad. Sci. USA.

[bib10] Dannenberg J.H., David G., Zhong S., van der Torre J., Wong W.H., Depinho R.A. (2005). mSin3A corepressor regulates diverse transcriptional networks governing normal and neoplastic growth and survival. Genes Dev..

[bib11] Eminli S., Utikal J., Arnold K., Jaenisch R., Hochedlinger K. (2008). Reprogramming of neural progenitor cells into induced pluripotent stem cells in the absence of exogenous Sox2 expression. Stem Cells.

[bib12] Engelen E., Akinci U., Bryne J.C., Hou J., Gontan C., Moen M., Szumska D., Kockx C., van Ijcken W., Dekkers D.H. (2011). Sox2 cooperates with Chd7 to regulate genes that are mutated in human syndromes. Nat. Genet..

[bib13] Fantes J., Ragge N.K., Lynch S.A., McGill N.I., Collin J.R., Howard-Peebles P.N., Hayward C., Vivian A.J., Williamson K., van Heyningen V., FitzPatrick D.R. (2003). Mutations in SOX2 cause anophthalmia. Nat. Genet..

[bib14] Fauquier T., Rizzoti K., Dattani M., Lovell-Badge R., Robinson I.C. (2008). SOX2-expressing progenitor cells generate all of the major cell types in the adult mouse pituitary gland. Proc. Natl. Acad. Sci. USA.

[bib15] Fero M.L., Rivkin M., Tasch M., Porter P., Carow C.E., Firpo E., Polyak K., Tsai L.H., Broudy V., Perlmutter R.M. (1996). A syndrome of multiorgan hyperplasia with features of gigantism, tumorigenesis, and female sterility in p27(Kip1)-deficient mice. Cell.

[bib16] Garcia-Lavandeira M., Quereda V., Flores I., Saez C., Diaz-Rodriguez E., Japon M.A., Ryan A.K., Blasco M.A., Dieguez C., Malumbres M., Alvarez C.V. (2009). A GRFa2/Prop1/stem (GPS) cell niche in the pituitary. PLoS ONE.

[bib17] Gleiberman A.S., Michurina T., Encinas J.M., Roig J.L., Krasnov P., Balordi F., Fishell G., Rosenfeld M.G., Enikolopov G. (2008). Genetic approaches identify adult pituitary stem cells. Proc. Natl. Acad. Sci. USA.

[bib18] Hong H., Takahashi K., Ichisaka T., Aoi T., Kanagawa O., Nakagawa M., Okita K., Yamanaka S. (2009). Suppression of induced pluripotent stem cell generation by the p53-p21 pathway. Nature.

[bib19] Kawamura T., Suzuki J., Wang Y.V., Menendez S., Morera L.B., Raya A., Wahl G.M., Izpisúa Belmonte J.C. (2009). Linking the p53 tumour suppressor pathway to somatic cell reprogramming. Nature.

[bib20] Kelberman D., Rizzoti K., Avilion A., Bitner-Glindzicz M., Cianfarani S., Collins J., Chong W.K., Kirk J.M., Achermann J.C., Ross R. (2006). Mutations within Sox2/SOX2 are associated with abnormalities in the hypothalamo-pituitary-gonadal axis in mice and humans. J. Clin. Invest..

[bib21] Kiyokawa H., Kineman R.D., Manova-Todorova K.O., Soares V.C., Hoffman E.S., Ono M., Khanam D., Hayday A.C., Frohman L.A., Koff A. (1996). Enhanced growth of mice lacking the cyclin-dependent kinase inhibitor function of p27(Kip1). Cell.

[bib22] Li H., Collado M., Villasante A., Strati K., Ortega S., Cañamero M., Blasco M.A., Serrano M. (2009). The Ink4/Arf locus is a barrier for iPS cell reprogramming. Nature.

[bib23] Marinoni I., Pellegata N.S. (2011). p27kip1: a new multiple endocrine neoplasia gene?. Neuroendocrinology.

[bib24] Marión R.M., Strati K., Li H., Murga M., Blanco R., Ortega S., Fernandez-Capetillo O., Serrano M., Blasco M.A. (2009). A p53-mediated DNA damage response limits reprogramming to ensure iPS cell genomic integrity. Nature.

[bib25] Martín A., Odajima J., Hunt S.L., Dubus P., Ortega S., Malumbres M., Barbacid M. (2005). Cdk2 is dispensable for cell cycle inhibition and tumor suppression mediated by p27(Kip1) and p21(Cip1). Cancer Cell.

[bib26] Masui S., Nakatake Y., Toyooka Y., Shimosato D., Yagi R., Takahashi K., Okochi H., Okuda A., Matoba R., Sharov A.A. (2007). Pluripotency governed by Sox2 via regulation of Oct3/4 expression in mouse embryonic stem cells. Nat. Cell Biol..

[bib27] Nakayama K., Ishida N., Shirane M., Inomata A., Inoue T., Shishido N., Horii I., Loh D.Y., Nakayama K. (1996). Mice lacking p27(Kip1) display increased body size, multiple organ hyperplasia, retinal dysplasia, and pituitary tumors. Cell.

[bib28] Pippa R., Espinosa L., Gundem G., García-Escudero R., Dominguez A., Orlando S., Gallastegui E., Saiz C., Besson A., Pujol M.J. (2012). p27(Kip1) represses transcription by direct interaction with p130/E2F4 at the promoters of target genes. Oncogene.

[bib29] Savatier P., Lapillonne H., van Grunsven L.A., Rudkin B.B., Samarut J. (1996). Withdrawal of differentiation inhibitory activity/leukemia inhibitory factor up-regulates D-type cyclins and cyclin-dependent kinase inhibitors in mouse embryonic stem cells. Oncogene.

[bib30] Sikorska M., Sandhu J.K., Deb-Rinker P., Jezierski A., Leblanc J., Charlebois C., Ribecco-Lutkiewicz M., Bani-Yaghoub M., Walker P.R. (2008). Epigenetic modifications of SOX2 enhancers, SRR1 and SRR2, correlate with in vitro neural differentiation. J. Neurosci. Res..

[bib31] Takahashi K., Yamanaka S. (2006). Induction of pluripotent stem cells from mouse embryonic and adult fibroblast cultures by defined factors. Cell.

[bib32] Taranova O.V., Magness S.T., Fagan B.M., Wu Y., Surzenko N., Hutton S.R., Pevny L.H. (2006). SOX2 is a dose-dependent regulator of retinal neural progenitor competence. Genes Dev..

[bib33] Tomioka M., Nishimoto M., Miyagi S., Katayanagi T., Fukui N., Niwa H., Muramatsu M., Okuda A. (2002). Identification of Sox-2 regulatory region which is under the control of Oct-3/4-Sox-2 complex. Nucleic Acids Res..

[bib34] Utikal J., Polo J.M., Stadtfeld M., Maherali N., Kulalert W., Walsh R.M., Khalil A., Rheinwald J.G., Hochedlinger K. (2009). Immortalization eliminates a roadblock during cellular reprogramming into iPS cells. Nature.

[bib35] Vandeva S., Vasilev V., Vroonen L., Naves L., Jaffrain-Rea M.L., Daly A.F., Zacharieva S., Beckers A. (2010). Familial pituitary adenomas. Ann. Endocrinol. (Paris).

[bib36] Williamson K.A., Hever A.M., Rainger J., Rogers R.C., Magee A., Fiedler Z., Keng W.T., Sharkey F.H., McGill N., Hill C.J. (2006). Mutations in SOX2 cause anophthalmia-esophageal-genital (AEG) syndrome. Hum. Mol. Genet..

[bib37] Zhao Y., Yin X., Qin H., Zhu F., Liu H., Yang W., Zhang Q., Xiang C., Hou P., Song Z. (2008). Two supporting factors greatly improve the efficiency of human iPSC generation. Cell Stem Cell.

