# Supplementary material for: p27Kip1 Directly Represses Sox2 during Embryonic Stem Cell Differentiation
Source: Cell Stem Cell. 2012 Dec 7;11(6):845–52. doi: 10.1016/j.stem.2012.09.014 (PMC3549496; doi:10.1016/j.stem.2012.09.014)
Supplement: Document S1. Figures S1–S4 and Supplemental Experimental Procedures [file mmc1.pdf]

Cell Stem Cell, volume *11*

Supplemental Information

p27^{Kip1} Directly Represses *Sox2* during Embryonic Stem Cell Differentiation

Han Li, Manuel Collado, Aranzazu Villasante, Ander Matheu, Cian J. Lynch, Marta Cañamero, Karine Rizzoti, Carmen Carneiro, Gloria Martínez, Anxo Vidal, Robin Lovell-Badge, and Manuel Serrano

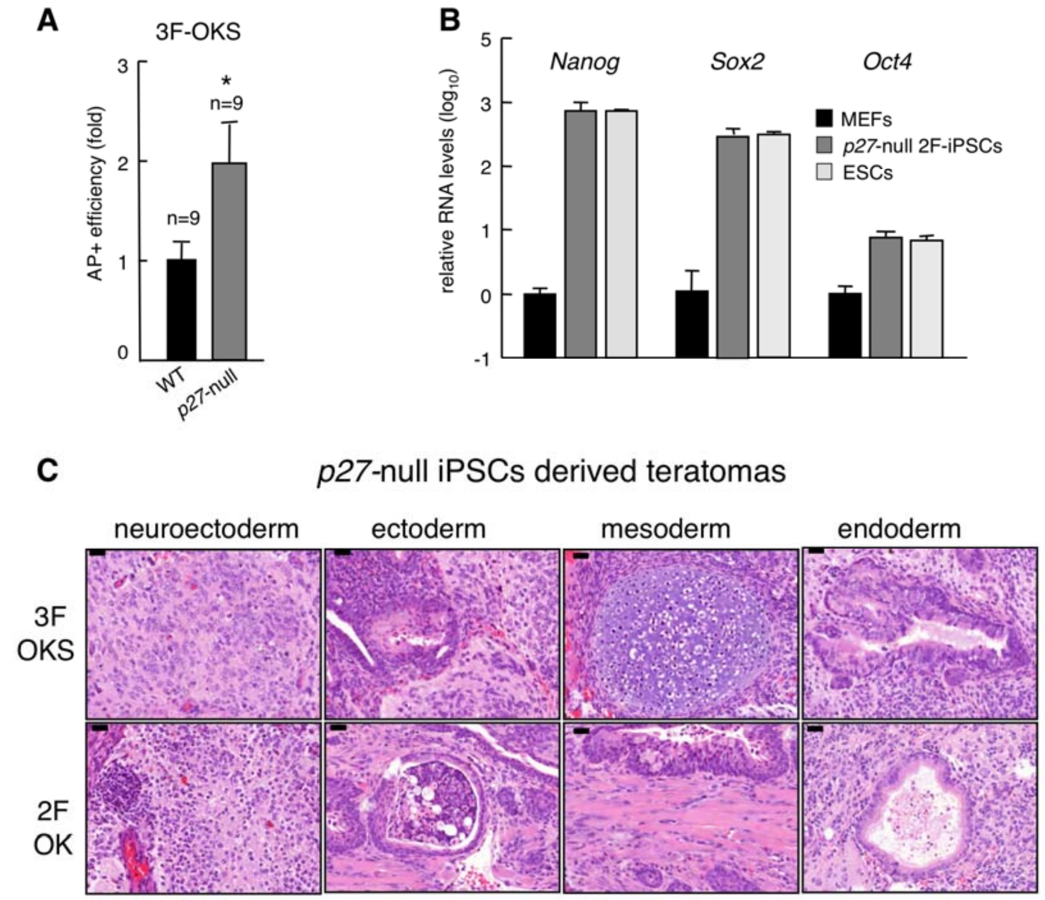

Figure S1. Relative reprogramming efficiency of *p27*-null MEFs by 3F-OKS and characterization of *p27*-null iPSCs, related to Figure 1.

(A) Relative average efficiency of generation of alkaline-phosphatase positive (AP^+) colonies produced by wt or *p27*-null MEFs transduced with *Oct4*, *Klf4* and *Sox2* (3F-OKS). Values correspond to the average ± s.d (n values indicate independent MEF isolates). Statistical significance was assessed by the two-tailed Student´s t-test: * $p < 0.05$.

(B) Stem cell markers expressed by *p27*-null 2F-OK iPSCs. Relative mRNA expression levels of *Nanog*, *Sox2* and *Oct4* in iPSCs obtained from *p27*-null MEFs reprogrammed by transduction of *Oct4* and *Klf4* (2F-OK). Embryonic stem (ESCs) cells and MEFs are shown as positive and negative controls, respectively. Values correspond to the average ± s.d.

(C) Histological analysis of teratomas induced by *p27*-null 3F-OKS iPSCs and by *p27*-null 2F-OK iPSCs. The indicated iPSCs (2×10^6 cells) were subcutaneously injected into irradiated (4 Gy) nude mice (injections were performed 1 day after irradiation). Teratomas were surgically removed after 3 weeks, fixed in 10% buffered formalin at 4 °C, embedded in paraffin wax, sectioned at a thickness of 5 µm, and stained with haematoxylin and eosin. Pathological examination revealed features of neuroectoderm, ectoderm, mesoderm and endoderm in 3F-OKS and in 2F-OK. The pictures are representative of three teratomas per condition, each teratoma derived from a different iPSC clone.

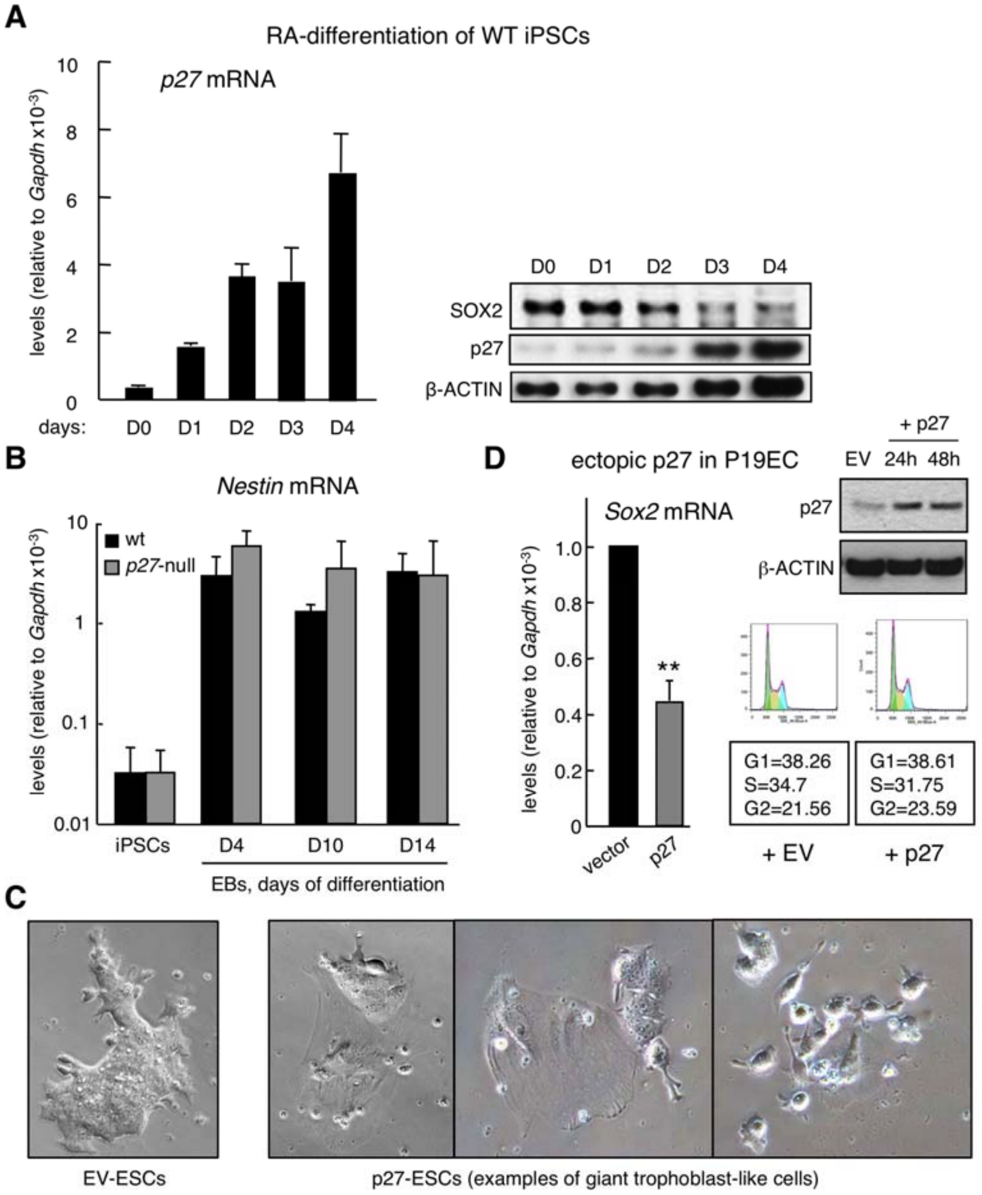

Figure S2. RA-differentiation in wt and *p27*-null iPSCs, related to Figure 2.

(A) Levels of *p27* mRNA and protein in wt iPSCs during RA-differentiation. *Left*, mRNA levels of 3 wt iPSC clones. *Right*, immunoblot of SOX2 and p27 at the indicated days of the differentiation protocol.

(B) Levels of *Nestin* mRNA in wt and *p27*-null embryoid bodies (EBs). Values correspond to 3 iPSC clones for each genotype (n=3). Differences were not significant.

(C) Giant trophoblast-like cells were observed in p27-overexpressing ESCs (3 days after retroviral infection with pMSCV-p27). These cells were not observed in ESCs infected with empty vector (EV-ESCs). Cells were plated on gelatin-coated plates.

(D) *Left*, levels of *Sox2* mRNA in P19EC cells 48 h after infection with an empty vector or with a plasmid overexpressing p27 (vector pBabe-puro). Values correspond to

two independent assays (n=2). *Top right*, overexpression of protein p27 was confirmed by immunoblotting. *Bottom right*, cell cycle profiles measured by propidium iodide indicated no difference in the proliferation rate of cells infection with empty vector (EV) or with p27.

Values correspond to the average ± s.d. Statistical significance was assessed by the two-tailed Student´s t-test: ** $p < 0.01$.

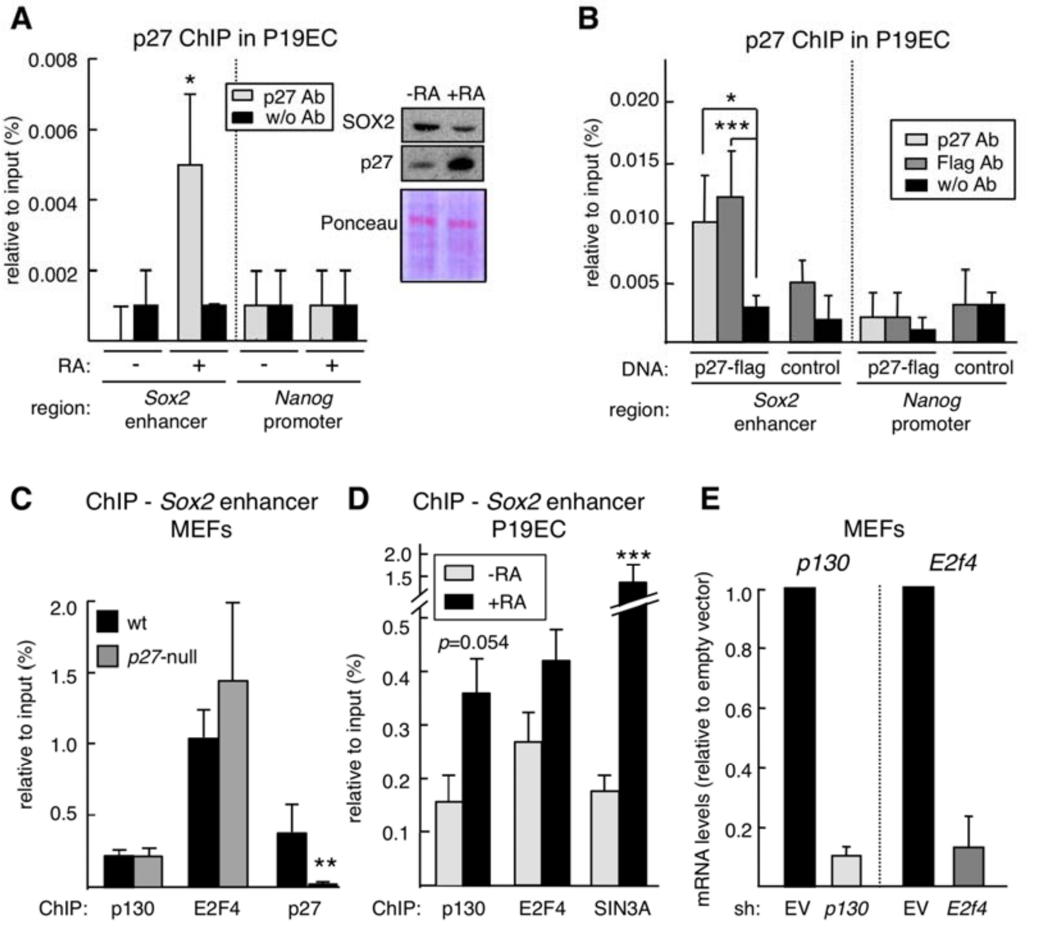

Figure S3. Binding of p27 and p130-E2F4-SIN3A to the *Sox2-SRR2* enhancer in differentiated P19EC cells, related to Figure 3.

(A) ChIP of p27 in the *Sox2-SRR2* enhancer and *Nanog* promoter before and after RA-differentiation using P19EC cells. Data correspond to one representative assay from a total of three independent assays. *Right*, protein levels of SOX2 and p27 were assessed by immunoblotting

(B) ChIP of p27 in P19EC cells 48 h after transfection with empty vector (control) or a plasmid expressing flag-tagged p27 (p27-flag).

(C) ChIP of p130, E2F4 and p27 on the *Sox2-SRR2* enhancer of MEFs.

(D) ChIP of p130, E2F4 and SIN3A on the *Sox2-SRR2* enhancer of P19EC cells before and after RA-differentiation.

(E) Downregulation of p130 and E2F4 after shRNA knockdown. Relative mRNA expression levels of p130 (left part of the graph) and E2F4 (right part of the graph) after shRNA knockdown (EV=empty vector). These samples correspond to the same samples shown in Figure 3G of the main manuscript.

Values correspond to the average ± s.d. Statistical significance was assessed by the two-tailed Student´s t-test: *** $p < 0.001$; * $p < 0.05$.

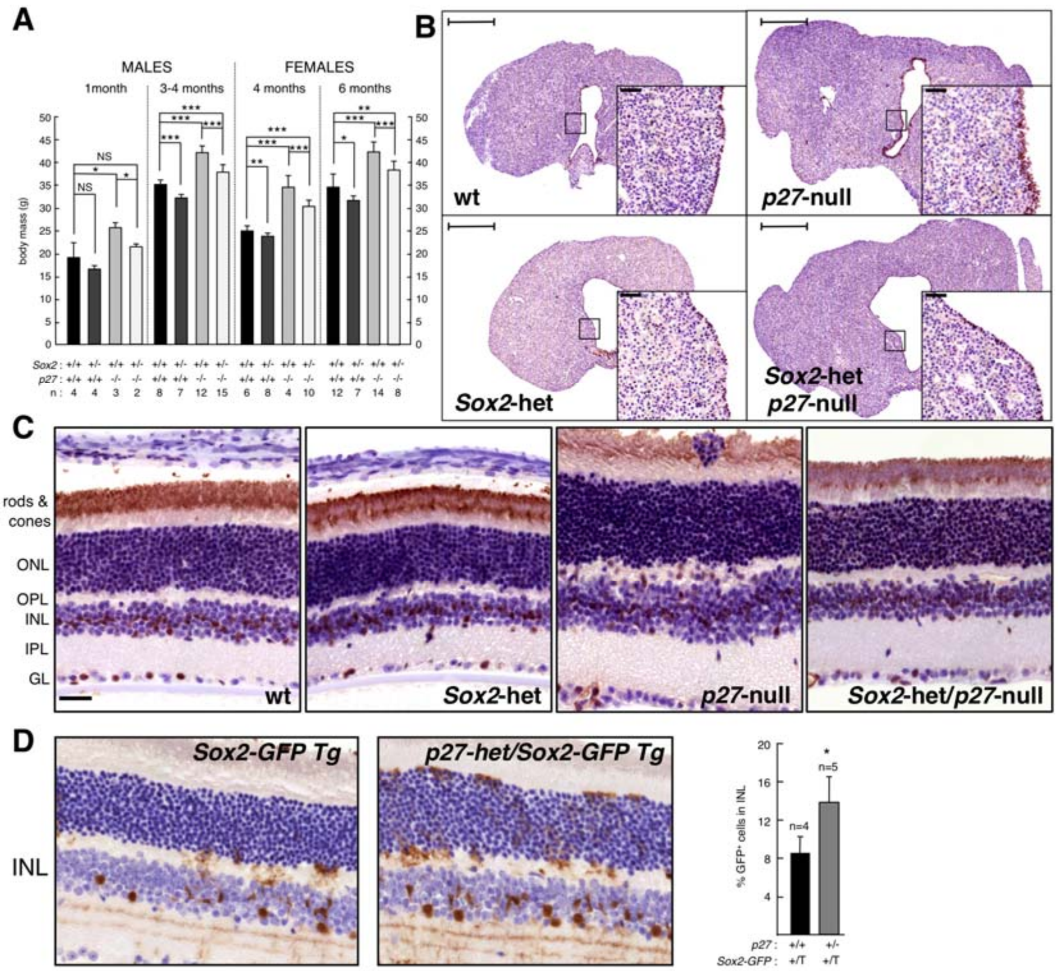

Figure S4. Increased body mass and retinal defects in *p27*-null mice are rescued by *Sox2*-haploinsufficiency, related to Figure 4.

(A) Average body mass of mice of the indicated sex, age and genotype.

(B) Low magnification picture of pituitaries of the indicated genotypes. Pituitaries were extracted by traction, thus leaving attached to the brain the intermediate and posterior lobes. Transversal sections were stained for SOX2. Bars in the low magnification pictures correspond to 500 µm, and in the insets to 50 µm.

(C) Representative pictures of retinal sections of the indicated genotypes stained for SOX2. Note mislocalized SOX2-positive nuclei in the outer plexiform layer (OPL) of the *p27*-null retina. ONL=outer nuclear layer; OPL= outer plexiform layer; INL= inner nuclear layer; IPL=inner plexiform layer; GL=granular layer. Bar on lower-left panel corresponds to 25 µm. Quantification of SOX2-positive nuclei in the entire retinal section is shown in the main Figure 4H.

(D) Representative pictures of retinal sections of the indicated genotypes stained for GFP. Note the increased abundance of GFP-positive nuclei in the inner nuclear layer (INL) of *p27*-heterozygous retinas. Quantification of GFP-positive nuclei in the INL is shown in the right panel.

Values correspond to the average ± s.d. Statistical significance was assessed by the two-tailed Student´s t-test: *** $p < 0.001$; ** $p < 0.01$; * $p < 0.05$.

SUPPLEMENTAL EXPERIMENTAL PROCEDURES

Mice

Mice *p27*-null (Fero et al., 1996), *Sox2*-heterozygous (Avilion et al., 2003), and *Sox2*-promoter/*GFP* transgenic (D'Amour and Gage, 2003) have been previously described. All comparisons were made among mice derived from the same sets of crosses, therefore sharing the same genetic background. Animal experimentation at the CNIO, Madrid, was performed according to protocols approved by the CNIO-ISCIII Ethics Committe for Research and Animal Welfare (CEIyBA). Animal experimentation at the MRC-NIMR, Mill Hill, London, was carried out in accordance with the UK Animals (Scientific Procedures) Act 1986.

Culture conditions

All the experiments with MEFs were performed at an early passage (passage 2-4). MEFs and P19EC cells were cultured in standard DMEM medium with 10% FBS (Gibco). For iPSC culture, cells were grown in DMEM (high glucose) supplemented with 15% serum replacement (KSR, Invitrogen), LIF 1000 u/ml, non-essential amino acids, glutamax and ß-mercaptoethanol (we refer to this below as "complete KSR medium"). ESCs were grown in the presence of 15% fetal bovine serum (and in the absence of KSR), all the other components were as in the medium for iPSCs. ESCs and iPSCs were routinely grown on feeder cells. For differentiation assays or retroviral infections, ESCs and iPSCs were previously adapted to grow on gelatin-coated plates (in the absence of feeder cells).

Generation of iPS cells

Reprogramming of primary (passage 2-4) mouse embryo fibroblasts was performed as previously described by us (Li et al., 2009) using plasmids pMXs-Klf4, pMXs-Sox2 or pMXs-Oct4 (obtained from Addgene and previously described (Takahashi and Yamanaka, 2006)). For quantification of alkaline phosphatase-positive (AP^+) efficiency, retroviral transduction was measured in parallel infections with a GFP retroviral supernatant added to the supernatant with reprogramming, and followed by FACS analysis at day 3 post-infection. The total number of AP^+ colonies was counted 3 weeks post-infection after staining plates for alkaline phosphatase activity (AP detection kit,

Chemicon International). Efficiency was calculated as the ratio between the number of AP^+ colonies and the number of GFP^+ cells.

Plasmids and infections

Retroviral vector pBabe-mouse-p27-puro was used for infection of primary MEFs and P19EC cells, and retroviral vector pMSCV-mouse-p27-puro for infection of ESCs. All the infections were carried out in the same manner. Briefly, 4 µg of DNA were transfected in HET 293 cells using Fugene HD according to the manufacturer's protocols. The target cells were infected twice per day (12 hours interval) for two days. After infection, cells were selected with puromycin (2ug/ml) for 3 days before harvesting for RNA and protein extraction. Flag-tagged mouse p27 was expressed from a pcDNA vector that was introduced into ESCs by nucleofection using the Neon transfection system (Invitrogen). In the case of P19EC cells, flag-tagged p27 was introduced using Fugene.

Gene silencing

The retroviral vector expressing shRNA for E2F4 gene silencing and the corresponding empty vector MSCV-LMP were generously provided by Jacqueline Lees (MIT, Boston, USA). Lentiviral plasmids for shRNA-mediated gene silencing of p130 were purchased from Sigma (TRCN0000071274, TRCN0000071276).

Differentiation with retinoic acid

Differentiation with retinoic acid (RA) was performed essentially as described (Savatier et al., 1996). ESCs or iPSCs were adapted to grow on gelatin-coated plates (and in the absence of feeder cells). Cells were grown to near confluency in their corresponding complete medium (day 0) and then were trypsinized and seeded at lower density in the absence of LIF for one day (day 1). During the following two days (days 2 and 3), RA was added at a concentration of 10^{-6} M and day 4 cells were without LIF and without RA. In the case of P19EC cells, differentiation was induced by addition of RA (10^{-6} M) for 4 days.

Embryoid body formation

iPSCs were grown on gelatin-coated plates for 3 passages to deplete mouse embryonic feeder fibroblasts. Once they reached 70% confluency passage, cells were trypsinized

and counted. After removing trypsin, iPSCs were resuspended in LIF-free KSR medium (see above) at a density of $2.5x10^5$/ml. Small volumes of 20 µl were plated as droplets on the lid of the Petri dish and an average 50 hanging drops were cultured over a cell culture dish containing PBS. After 3 days, droplets were collected and transferred to a Petri dish and further cultured in LIF-free KSR medium for 10 days before harvested for qRT-PCR analysis.

RNA analysis

Total RNA was extracted with Trizol (Life Technologies). Samples were treated with DNAseI before reverse transcription using random priming and Superscript Reverse Transcriptase (Life Technologies), according to the manufacturer's protocols. Quantitative real-time PCR was performed using an ABI PRISM 7700 (Applied Biosystems) and DNA Master SYBR Green I mix (Applied Biosystems). Calculation for the values were made using the ΔΔCt method (Yuan et al., 2006). The primers used were:

Brachyury-F	5’-CTGCGCTTCAAGGAGCTAAC-3’
Brachyury-R	5’-CCAGGCCTGACACATTTACC-3’
Cdx2-F	5’-CCGCAGAACTTTGTCAGTCCT-3’
Cdx2-R	5’-GTAACCACCGTAGTCCGGGTA-3’
Csh1-F	5’-GGTGTCAAGCCTACTCCTTTG-3’
Csh1-R	5’-GTATTATGGAGCAGTTCAGCCAA-3’
Eomes-F	5’-GCGCATGTTTCCTTTCTTGAG-3’
Eomes-R	5’-GGGGTTGAGTCCGTTTATGTT-3’
Fgf5-F	5’-TGTACTGCAGAGTGGGCATC-3’
Fgf5-R	5’-ACAATCCCCTGAGACACAGC-3’
Gapdh-F	5’-TTCACCACCATGGAGAAGGC-3’
Gapdh-R	5’-CCCTTTTGGCTCCACCCT-3’

Gata4-F	5'-CCCTACCCAGCCTACATGG-3'
Gata4-R	5'-ACATATCGAGATTGGGGTGTCT-3'
Nanog-F	5'-AGGGTCTGCTACTGAGATGCTCTG-3'
Nanog-R	5'-CAACCACTGGTTTTTCTGCCACCG-3'
Nestin-F:	5'-CCCTGAAGTCGAGGAGCTG-3'
Nestin-R:	5'-CTGCTGCACCTCTAAGCGA-3'
p27-F	5'-TCAAACGTGAGAGTGTCTAACG-3'
p27-R	5'-CCGGGCCGAAGAGATTTCTG-3'
Sox2-F:	5'-TAGAGCTAGACTCCGGGCGATGA-3'
Sox2-R:	5'-TTGCCTTAAACAAGACCACGAAA-3'

Chromatin immunoprecipitation

Cells were crosslinked with 1% formaldehyde for 15 min at room temperature. Crosslinking was stopped by the addition of glycine to a final concentration of 0.125 M. Fixed cells were lysed in lysis buffer (1% SDS, 10 mM EDTA, 50 mM Tris-HCl, pH 8.0) and sonicated. An aliquot of 60 µg was reserved as input. For immunoprecipitation, 600 µg of protein were diluted in dilution buffer (1% Triton X-100, 2 mM EDTA, 150 mM NaCl and 20mM Tris-HCl, pH 8.0, containing protease inhibitors), and pre-cleared with A/G plus-agarose (SantaCruz). The antibodies used for the immunoprecipitation were histone H3 trimethyl-Lys27 (Upstate), histone H3 trimethyl-Lys9 (Upstate), p27 (Santa Cruz, C-19), p130 (Santa Cruz, C-20), E2F4 (Santa Cruz, A-20), SIN3A (Upstate), Flag (Sigma, F1804). Immune complexes were precipitated with A/G plus-agarose and washed sequentially with low-salt im mune complex wash buffer (0.1% SDS, 1% Triton X-100, 2 mM EDTA, 20 mM Tris-HCl, pH 8.1, 150 mM NaCl), high-salt immune complex wash buffer (0.1% SDS, 1% Triton X-100, 2 mM EDTA, 20 mM Tris-HCl, pH 8.1, 500 mM NaCl), LiCl immune complex wash buffer (0.25 M LiCl, 1% NP-40, 1% deoxycholate-Na, 1 mM EDTA, 10 mM Tris-HCl, pH 8.1), and TE buffer, and finally eluted in elution buffer (1% SDS, 0.1 M $NaHCO_3$). All samples, including inputs, were de-crosslinked, treated with proteinase

K, and DNA was extracted with phenol–chloroform and resuspended in TE buffer. All PCR reactions were carried out in triplicate.

The primers used for PCR after ChIP were:

Nanog-promoter-F:	5'-CAACTTACTAAGGTAGCCCGAGTCTTAA-3'
Nanog-promoter-R:	5'-CCTCCAAAAGTGCGGCTTT-3'
Sox2-SRR2-F:	5'-ATTTATTCAGTTCCCAGTCCAAGC-3'
Sox2-SRR2-R:	5'-CCCTCTCCCCCCACGC-3'

Immunohistochemistry

Tissues were fixed in 10% buffered formalin at 4°C, embedded in paraffin wax, and sectioned at a thickness of 5 mm. Sections were stained with hematoxylin and eosin for pathological examination or processed for immunohistochemical analysis with antibodies against SOX2 (Millipore, AB5603) or against GFP (Roche, clones 7.1 and 13.1). After staining, whole retina and pituitary sections were scanned with Mirax Scan (Zeiss), and visualized with Pannoramic Viewer and quantified using Nuclear Quant (both from 3DHisTech). Counting was performed on representative fields at the same magnification, on a minimum of 3 different areas per sample, and a minimum of 3 different samples per genotype.

Confocal analysis

MEFs were seeded in cover slips the day before ($1x10^5$/slide). Cells were fixed in 4% paraformaldehyde for 20 minutes, permeabilized (PBS-0.1% Triton X-100) for 15 minutes and blocked (PBS-5% BSA) for one hour in room temperature. Antibodies for SOX2 (Chemicon, AB5603, 1:500) and p27 (Neomarker, DCS-72.F6, 1:250) were added and incubated in PBS-2%BSA overnight. Next day, cells were washed with PBS and incubated with secondary antibody (anti-rabbit Cy3, 1:500; anti-rabbit Alexa 488 chicken, 1:200; anti-mouse Alexa 555 donkey, 1:200) followed by counterstaining with DAPI (10mg/ml, Sigma). Cells were inspected under a Leica TCS-SP5 confocal microscope (AOBS) and analyzed using Definiens Developer XD 1.5 software, under the same exposure conditions.

SUPPLEMENTAL REFERENCES

Avilion, A.A., Nicolis, S.K., Pevny, L.H., Perez, L., Vivian, N., and Lovell-Badge, R. (2003). Multipotent cell lineages in early mouse development depend on SOX2 function. Genes Dev *17*, 126-140.

D'Amour, K.A., and Gage, F.H. (2003). Genetic and functional differences between multipotent neural and pluripotent embryonic stem cells. Proc Natl Acad Sci U S A *100 Suppl 1*, 11866-11872.

Fero, M.L., Rivkin, M., Tasch, M., Porter, P., Carow, C.E., Firpo, E., Polyak, K., Tsai, L.H., Broudy, V., Perlmutter, R.M., et al. (1996). A syndrome of multiorgan hyperplasia with features of gigantism, tumorigenesis, and female sterility in p27(Kip1)-deficient mice. Cell *85*, 733-744.

Li, H., Collado, M., Villasante, A., Strati, K., Ortega, S., Canamero, M., Blasco, M.A., and Serrano, M. (2009). The Ink4/Arf locus is a barrier for iPS cell reprogramming. Nature *460*, 1136-1139.

Savatier, P., Lapillonne, H., van Grunsven, L.A., Rudkin, B.B., and Samarut, J. (1996). Withdrawal of differentiation inhibitory activity/leukemia inhibitory factor up-regulates D-type cyclins and cyclin-dependent kinase inhibitors in mouse embryonic stem cells. Oncogene *12*, 309-322.

Takahashi, K., and Yamanaka, S. (2006). Induction of pluripotent stem cells from mouse embryonic and adult fibroblast cultures by defined factors. Cell *126*, 663-676.

Yuan, J.S., Reed, A., Chen, F., and Stewart, C.N., Jr. (2006). Statistical analysis of real-time PCR data. BMC Bioinformatics *7*, 85.
